# The prevalence of functional disability and associated factors among older adults living in Bahir Dar City, Northwest, Ethiopia: a community-based cross-sectional study

**DOI:** 10.3389/fpubh.2024.1399027

**Published:** 2024-10-10

**Authors:** Addisu Yihenew, Yeshambel Ejigu Anteneh, Tewodros Miheret, Melese Gobezie, Getachew Azeze Eriku, Yadelew Yimer Shibabaw, Tesfa Kassa

**Affiliations:** ^1^Department of Physiotherapy, Bahir-Dar University, Bahir-Dar, Ethiopia; ^2^Department of Physiotherapy, School of Medicine, College of Medicine and Health Science, University of Gondar, Gondar, Ethiopia; ^3^Department of Biochemistry, School of Medicine, College of Medicine and Health Science, University of Gondar Comprehensive Specialized Hospital, Gondar, Ethiopia

**Keywords:** older adults, activities of daily living, functional disability, Bahir-Dar city, Ethiopia

## Abstract

**Background:**

The limitation of carry out everyday activities independently is a common experience for older adults with functional disabilities, which can hurt their overall well-being. Unfortunately, there is still a dearth of evidence about how common it is among older adults, especially in low-and middle-income countries like Ethiopia. Therefore, the study aimed to assess the prevalence and associated factors of functional disability among older adults living in Bahir Dar city, Northwest, Ethiopia, 2023.

**Method:**

A community-based cross-sectional study was conducted among older adults (≥60 years) living in Bahir Dar city, Northwest, Ethiopia. A total of 598 study participants were included using a multistage sampling technique. Data were collected through interviewer-administered questionnaires. Barthel Index (BI) was used to assess the functional capacity of older adults which comprises 10 items. The total score ranges from 0 to 100. Variables significant in bivariable analysis were entered into multivariable logistic regression analysis. A *p* < 0.05 was considered statistically significant in the final logistic regression model.

**Results:**

Among the 598 participants studied, the overall prevalence of functional disability was 29.6 (95% CI: 26.0–33.4). Older age (≥70 years) {adjusted odds ratio(AOR: 2.24; 95% CI: 1.03–4.88)}, comorbidity (AOR: 2.96; 95% CI: 1.47–5.94), physically inactive (AOR: 2.42; 95% CI: 1.18–4.96), one and more drug users (AOR: 3.61; 95% CI: 2.12–6.15), cognitive impairment (AOR: 3.66; 95% CI: 2.26–4.93) and depression (AOR: 1.70; 95% CI: 1.08–2.68) were found significant associated factors of functional disabilities among older adults.

**Conclusion:**

This study found that nearly one-third of older adults had functional disabilities. Functional disability was significantly associated with factors such as increasing age, presence of comorbidities, being physically inactive, one and more drug users, who developed cognitive impairment, and having depression symptoms. Proactive measures need to be initiated to ensure proper care and support of functionally disabled individuals, especially targeting vulnerable groups identified in this study.

## Introduction

According to demographic estimates, every country’s population is expected to age, which will have a significant impact on the social, economic, and health sectors (1). While many older adults lead active lives and make valuable contributions to their local communities and wider society, they frequently report functional disabilities. The most typical way to test this is by looking at how well an older adult can do instrumental activities of daily living (IADL) like cooking a hot meal, grocery shopping, or taking medication, as well as activities of daily living (ADL) like getting dressed, walking across a room, and bathing (2).

Even though functional disability is a significant public health concern, little is known about how common it is among older adults, particularly in low-and middle-income nations. According to a World Health Organization (WHO) report, 15% of the world’s older population is functionally disabled (3). The prevalence of functional disabilities in high-income and low-income countries was 29.5 and 43.4%, respectively (4). Based on the Global Health Survey report, functional disability was primarily seen in those aged ≥70 years in the United States, Netherlands, Sweden, and Switzerland countries (1, 5, 6). In Europe, the prevalence of at least one ADL disability ranged between 11 and 44% (7). However, there is a dearth of evidence in low-income countries like Ethiopia that shows the magnitude of functional disability among older adults.

Older adults with a functional disability are more likely to have poor social networks, less engagement in work, poor health, dependence on ADL, increased healthcare costs, and greater mortality (4, 8). Thus, functional disability lowers the quality of life and functional independence of older adults (9). Moreover, the presence of functional decline may increase the demand for resources for medical and rehabilitation care or admission to residential care and may result in premature death (10). Such a community-based comprehensive study of functional disability in older adults is a fundamental crucial aspect of providing care, understanding the burden of functional disability, and predicting the need for assistance for this age group (11).

According to previously published studies, chronic diseases, age, sex, socioeconomic position, physical activity, BMI, educational attainment, smoking, depression, and history of hospitalization were significantly associated factors with functional disability (2, 12–14). However, those identified associated factors were differentiated through studies conducted in high-income countries. Therefore, the investigation of those factors in low-income countries like Ethiopia among older adults will be very helpful in providing high-quality care and social services (15). To the best of our knowledge, no study has investigated the burden and associated factors of functional disability among older adults in Bahir Dar city, Ethiopia. Therefore, this study aimed to determine the burden and associated factors with functional disability among older adults in Bahir-Dar city, Northwest Ethiopia.

## Materials and methods

### Study design, period, and setting

A community-based cross-sectional study was conducted from April to May 2023 among older adults to determine the prevalence of functional disabilities and the potential associated factors.

The study was conducted in Bahir Dar City, the capital of Amhara regional state, located in Northwestern Ethiopia, 565 kilometers from Addis Abeba. According to the Bahir Dar city administration’s Bureau of Labor and Social Affairs statistics, the total population of the city is estimated to be 390,429. The city has six sub-cities administration and a total of 26 kebeles (i.e., the local administration level next to woredas/sub-cities). The total number of older adults (≥60 years) living in the city was 11,034. Each older adult’s name, age, sex, and full address were included in the profile. Both male and female older adults aged 60 years and older permanently residing six and above months in the selected kebeles were included in the study. On the other hand, older adults who were severely ill and could not respond were excluded from the study. The city now has two comprehensive specialized hospitals that provide healthcare to the surrounding population from the West Gojjam, East Gojjam, Awi, south Gondar, and central Gondar zones.

### Sample size determination and sampling technique

The required sample size was determined by using the single population proportions formula, using the assumption of 50% prevalence since there is no previously published study with a similar population, a 95% confidence interval, and a 5% margin of error.


$$N=\;\frac{\left(z\;\left(\frac{\alpha }{2}\right)2\;\left(p\right)\left(1-p\right)\right)}{d^{2}}=385$$

Since the study used a multistage sampling technique by considering a design effect of 1.5 and 10% non-response rate the final sample size was 636.

**Figure 1:**
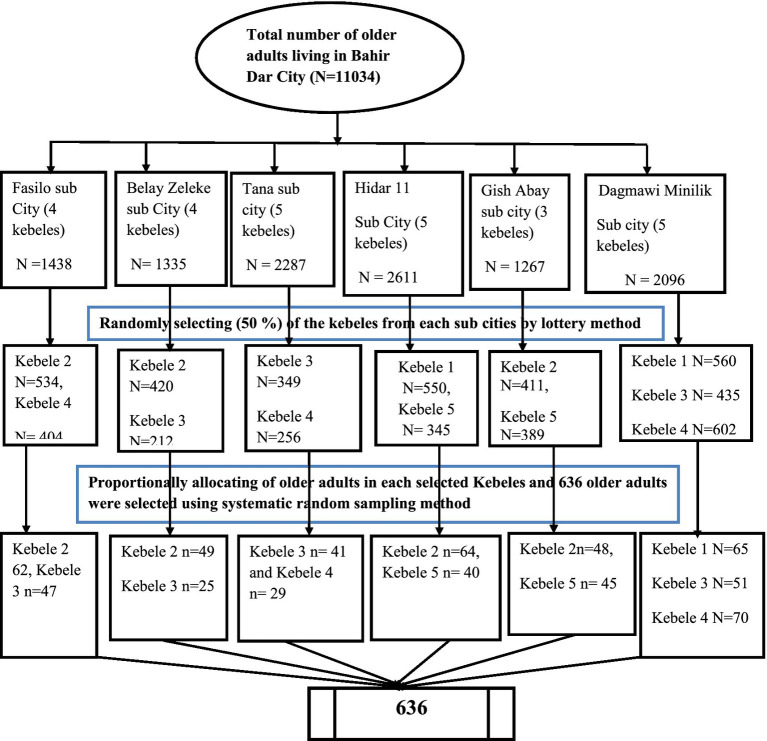
Schematic presentation of sampling procedure among older adults living in Bahir Dar city, 2023.

A multistage sampling technique was used to select the study participants. Bahir Dar city has 6 sub cities and 26 kebeles. A total of 13 kebeles (50 percent of the total kebeles) from each sub-city were selected by a simple random sampling technique to obtain an adequate final sample size. Subsequently, proportionally allocate the total sample size from the selected kebeles. Finally, a systematic random sampling technique was used to select the study participants in each kebele based on the full address list of participants. All older people living in the selected kebeles registered their full addresses, such as age, sex, and house number. We calculated the *K* value by dividing the total older adults in each selected kebele into proportional sample sizes for each selected kebele, and it was eight (*k* = 8). We interviewed all eligible older adults in the selected kebeles in their households based on the house number. An initial older adult was randomly selected by a lottery method. Another older adult was then selected from the initial order at every Kth interval. Whenever more than one eligible older adult was found in the same selected household, only one of them was selected by lottery method (Figure 1).

### Operational definition

Functional disability: measured by the Barthel index (BI) of independence in activities of daily living, if a subject met at least one of these 10 ADL problems, they were classified as having a functional disability in ADL or with a score of ≤95 (11, 16).

Older people: the ages at which a person reaches ≥60 years of age were considered to be older adults (11).

Cognitive impairment: in the mini-mental state examination (MMSE), participants with scores ≤24 out of a total of 30 scores had cognitive impairment (17).

Depression: measured by the geriatric depression scale (GDS) short form, an individual with a score of ≥5 was considered to be depressed (18).

Physical activities: any kind of moderate-intensity exercise (such as walking, cycling, sports, or planned exercise and strength exercise) done at least 150 min per week (19).

Body mass index (BMI): underweight (<18 kg/m^2^), normal (18–24.99 kg/m^2^), overweight (≥25.00–29.9 kg/m^2^), and obese (30.0 kg/m^2^ and above) (20).

Alcohol consumption: alcohol consumers were simply defined as older adults who were drinking beer, areke, Tella, or tej every day or every other day (21).

Hospitalization: it was defined as participants with a history of hospitalization for the last 12 months considered as a hospitalization.

Comorbidity: it was defined as when a participant had one or more associated medical problems such as diabetes mellitus, hypertension, and others.

Smoker: current smoker: someone who had smoked more than 100 cigarettes in their lifetime and now, who smoked every day; previous smoker: someone who had smoked more than 100 cigarettes in their lifetime but had not smoked in the last 28 days (22).

### Data collection tool and procedure

The data were collected using a five-section questionnaire and structured interviews with participants. The questionnaire comprises sociodemographic variables (age, sex, marital status, body mass index, educational level, living arrangement, and income status), health-related questionnaires (hospitalization, one and more drug users, comorbidity, cognitive impairment), psychological factors (depression), and lifestyle factors (smoking, alcohol consumption, and physical activity), and data on functional disability using the BI. BI has been used widely for the assessment of the functional capacity of older adults which contains 10 items comprised of feeding, bathing, grooming, dressing, bowels continent, bladder continent, toilet use, transfer(bed to chair and back), mobility(on level surface) and stairs climbing and down with a total score of 100. Two items are rated on 0 (unable) and 5(independent). On the other hand, six items are rated with 0(dependent), 5(needs help), and 10(independent). Moreover, two items are rated with 0 (unable), 5(major help), 10(minor help), and 15(independent). Among older participants, if he/she had dependency in at least one of these 10 ADLs or with a score of ≤95, he/she was considered functionally disabled (11, 16). To measure weight and height, we used the weight scale tool specification TIANSHA = 2003A, capacity: 11 lb./5 kg–400 lb./180 kg, Battery: CR2032 lithium battery to measure (23). We also measured height using the tape measure tool specification WIN TAPE, (FT-070) (2). For data collection, the questionnaire was translated from the English version to the Amharic version and translated back to English to ensure its consistency. Older adults who were willing to participate in the study were screened using the inclusion and exclusion criteria. Data collectors interviewed the participants after obtaining informed consent. Data were collected by five trained BSC physiotherapists supervised by two senior physiotherapists.

### Data quality control and analysis

To ensure the quality of the data, the principal investigator trained the data collectors and supervisors on how to approach the participants, the objective of the study, and ethical issues 1 day before the data collection began. The supervisor checked for the completeness and consistency of the data. To check for the accuracy of responses, language clarity, and appropriateness of the tools, the questionnaire was pre-tested on 5% of the sample size. Before the actual data collection period, data were collected on a population other than the study population, which has characteristics similar to those populations selected for the actual study. It has been pre-tested in Gondar kebele 16 before the actual data collection. However, no necessary changes were made in the actual study based on the findings of the pre-test. Data were checked for completeness, accuracy, and clarity by the principal investigator before data entry. Then, the data were coded and entered into Epidata version 4.6 and exported to SPSS Software version 25 for data analysis. Descriptive analysis was done for all variables and numerical summary measures, frequency distribution tables, and graphs were used to describe the study population about relevant variables. The chi square test (*χ*2) of each variable was checked before factor analysis. Multicollinearity was checked by the variance inflation factor (VIF < 10) and model fitness was checked by the Hosmer-Lemeshow goodness of fit test. Binary logistic regression analysis was employed to determine predictors of the outcome variable. A factor with a *p*-value less than 0.2 was selected for multivariable analysis. Whereas, variables those had two sided *p*-value less than 0.05 were considered factors associated with functional disability in the multivariable logistic regression analysis.

## Results

### Socio-demographic, clinical, lifestyle-related and psychological characteristics of the participants

Five hundred ninety eight older adults participated in this study, with a response rate of 94.02% of the total sample size. The reasons for non-responses were due to time constraints and lack of interest. Among the total respondents, more than half of 330 (55.2%) were female. The age of respondents ranged from 61–90 years, with a mean age and standard deviation of 69.43 ± 7.2 years. The majority of the participants 266 (44.3%) were married. Nearly one-third of the participants, 190 (31.8%), had completed primary school, more than half of them, 317(53.0%) lived with children or other family members, and nearly one-fourth of the study participants 144 (24.1%) were overweight. More than one-fourth of the participants 161 (26.9%) were pharmacy users, and 197 (32.9%) had a history of hospitalization in the past year. Five hundred seventeen (86.5%) of the total participants engaged in physical activity less than 150 min per week (Table 1).

**Table 1:** Socio-demographic, clinical, lifestyle-related and psychological variables of the study participant among older adults living in Bahir Dar City, Northwest Ethiopia, 2023 (*n* = 598).

Variables	Categories	Sample	Functional disability *n* (%)		*χ*2	*p*-value
		*n* (%)	No	Yes		
Gender	Male	268 (44.8)	186 (44.2)	82 (46.3)	0.2	0.63
	Female	330 (55.2)	235 (55.8)	95 (53.7)		
Age (years)	60–64	119 (19.9)	95 (22.6)	24 (13.6)	57.8	0.001
	65–69	355 (59.4)	273 (64.8)	82 (46.3)		
	≥70	124 (20.7)	53 (12.6)	71 (40.1)		
Educational status	Unable to read and write	107 (17.9)	69 (16.4)	38 (21.5)	3.4	0.34
	Primary school	190 (31.8)	141(33.5)	49 (27.7)		
	Secondary school	167 (27.9)	119 (28.3)	48 (27.1)		
	Collage and above	134 (22.4)	92 (21.9)	42 (23.7)		
Income status(ETB)	<1,500	112 (18.7)	76 (18.1)	36 (20.3)	1.1	0.57
	1,500–3,500	132 (22.1)	90 (21.4)	42 (23.7)		
	>3,500	354 (59.2)	255 (60.6)	99 (55.9)		
Marital status	Married	266 (44.5)	220 (52.3)	46 (26.0)	35.1	0.001
	Divorce	154 (25.7)	91 (21.6)	63 (35.6)		
	Widow	178 (29.8)	110 (26.1)	68 (38.4)		
Living arrangement	Living alone	106 (17.7)	71 (16.9)	35 (19.8)	1.2	0.54
	Living with children/family	317 (53.0)	222 (52.7)	95 (53.7)		
	Living with spouse only	175 (29.3)	128 (30.4)	47 (26.6)		
Body mass index (BMI)	Normal	423 (70.7)	302 (71.7)	121 (68.4)	0.9	0.64
	Under Weight	31 (5.2)	20 (4.8)	11 (6.2)		
	Overweight	144 (24.1)	99 (23.5)	45 (25.4)		
Drug users	No	437 (73.1)	356 (84.6)	81 (45.8)	95.3	0.001
	Yes	161 (26.9)	65 (15.4)	96 (54.2)		
Comorbidity	No	528 (88.3)	399 (94.8)	129 (72.9)	57.8	0.001
	Yes	70 (11.7)	22 (5.2)	48 (27.1)		
Hospitalization	No	401 (67.1)	305 (72.4)	96 (54.2)	18.7	0.001
	Yes	197 (32.9)	116 (27.6)	81 (45.8)		
Depression	No	385 (64.4)	309 (73.4)	76 (42.9)	50.4	0.001
	Yes	213 (35.6)	112 (26.6)	101 (57.1)		
Cognitive impairment	No	413 (69.1)	343 (81.5)	70 (39.5)	102.5	0.001
	Yes	185 (30.9)	78 (18.5)	107 (60.5)		
Physical activity	No	517(86.5)	356 (84.6)	161 (91.0)	4.4	0.04
	Yes	81 (13.5)	65 (15.4)	16 (9.0)		
Smoker	No	569 (95.2)	402 (95.5)	167 (94.4)	0.4	0.56
	Yes	29 (4.8)	19 (4.5)	10 (5.6)		
Alcoholic	No	496 (82.9)	347 (82.4)	149 (84.2)	0.3	0.60
	Yes	102 (17.1)	74 (17.6)	28 (15.8)		

### Characteristics of older adults with functional disability

The overall prevalence of functional disability in this study was found to be 177 (29.6%) (95% CI 26.0–33.4). Among those, 95 (53.7%) female participants and 82 (46.3%) aged 65–69 years had functional disabilities. Compared to participants without functional disability, nearly half of the secondary and primary school participants reported majority of functional disability, 48 (27.1%) and 49 (27.7%), respectively. Furthermore, the majority of older adults who had functional disabilities were participants who lived with their family 95 (53.7%) compared to participants who lived alone 35 (19.8%) and spouses 47 (26.6%) (Table 1).

### Logistic regression model of factors associated with functional disability

After fitting a bivariable logistic regression model, factors such as age, marital status, comorbidity, hospitalization, cognitive impairment, physical exercise, one or more drug users, and people who develop depression are found with a *p*-value of <0.2. Finally, multivariable logistic regression analysis was fitted to control potential cofounders and to identify potential associated factors. In this model, factors such as age 70 and above (*p* = 0.041), being physically inactive (*p* = 0.015), having comorbidities (*p* = 0.002), being drug users (*p* = 0.001), having cognitive impairment (*p* = 0.001), and having depression (*p* = 0.022) were significantly associated with functional disability in multivariable logistic regression analysis (*p* < 0.05). Multicollinearity was checked by the VIF and it was<10. Model fitness was checked by the Hosmer-Lemeshow goodness of fit test and it had a *p*-value of 0.66.

The odds of being functional disability in participants aged 70 years and above were 2.24 times more likely to develop functional disability than those older adults who were 60–64 years old {adjusted odds ratio (AOR = 2.24, 95% CI 1.03–4.88)}. Participants who have comorbidities were 2.96 times more likely to develop functional disability than those with no comorbidities (AOR = 2.95, 95% CI 1.47–4.94). Similarly, those who have cognitive impairment were 3.66 times more likely to develop functional disability as compared with those who did not develop cognitive impairment (AOR = 3.66, 95% CI 2.26–4.93). Furthermore, older adults who developed depression symptoms were 1.7 times more likely to develop functional disability (AOR = 1.7, 95% CI 1.08–2.67) than those who did not develop depression symptoms. In addition to this, one or more drug user participants were 3.61 times more likely to develop functional disability than those who did not use any drug (AOR = 3.61, 95% CI 2.12–6.16). Likewise, for those physically inactive respondents, the odds of developing functional disability were 2.42 times higher as compared with those who were physically active (AOR = 2.42, 95% CI 1.18–4.96) (Table 2).

**Table 2:** Bivariable and multivariable logistic regression factors analysis of functional disabilities among older adults living in Bahir Dar City, Northwest Ethiopia 2023(*n* = 598).

Variables	Categories	Functional disability		COR (95% CI)	AOR 95%CI
		Yes	No		
Age (years)	60–64	24	95	1	1
	65–69	82	273	1.19(0.71–1.98)	0.82(0.44–1.54)
	≥70 and older	71	53	5.30(2.99–9.39)	2.24(1.03–4.96)*
Marital status	Married	46	220	1	1
	Divorce	63	91	3.31(2.11–5.20)	1.32(0.66–3.08)
	Widowed	68	110	2.96(1.90–4.58)	1.10(0.59–2.04)
Physical activities	Yes	16	65	1	1
	No	161	356	1.84(1.03–3.27)	2.42(1.18–4.96)*
Co-morbidity	Yes	48	22	6.75(3.92–11.60)	2.95(1.47–5.94)**
	No	129	399	1	1
Hospitalization	Yes	81	116	2.22(1.54–3.19)	0.91(0.55–1.49)
	No	96	305	1	1
Cognitive impairment	Yes	107	78	6.72(4.55–9.91)	3.66(2.26–5.93)**
	No	70	343	1	1
One and more drug users	Yes	96	65	6.49(4.36–9.65)	3.61(2.12–6.16)**
	No	81	356	1	1
Depression	Yes	101	112	3.67(2.53–5.29)	1.70 (1.08–2.69)*
	No	76	309	1	1

1 = Reference category, CI = confidence interval, COR = crude odds ratio, AOR = adjusted odds ratio, * = *p*-value < 0.05, and ** = *p*-value < 0.01.

## Discussion

The prevalence of functional disability among older adults living in Bahir Dar city was 29.6% (CI 95%, 26.0–33.4), which revealed that functional disability is a major public health concern among older adults in Bahir Dar city, Ethiopia. This suggests that in order to reduce the functional incapacities of older adults in the community, health care planning and delivery are essential. It is in line with a study conducted in Nigeria (28.3%) (24). This similarity may be due to using similar study designs and comparable mean ages of study participants. For example, the study carried out in Nigeria employed a multistage sampling technique to recruit participants and a community-based cross-sectional study design, just like our study. On the other hand, the majority of the participants had only received primary education, which may have a similar understanding of self-care and various health conditions that affect the functional level of older adults. Nearly the same proportion of participants in both groups reported having some chronic diseases that may lower their functional levels. Furthermore, since institutional care for the older people is exceedingly rare and the majority of the older adults live in the community with other family members, which may yield equivalent results, older adults in Ethiopia and Nigeria should be cared for by their family members.

However, the prevalence of this study was higher compared to studies conducted in Uberaba, Brazil (21.2%) (13), Lebanon (24%) (25), and Japan (20.1%) (14). One reason why our results and the Brazil study might differ is that the functional disability in the Brazil study was assessed using the six-item Katz Index instrument. On the other hand, the ten-items BI was employed in our study to assess functional disability. BI has been reported to be more sensitive in detecting functional disability as compared with the Katz Index. Additionally, older adults with cognitive deterioration were generally excluded from Brazil’s study based on the results of the MMSE assessment. They were nevertheless included in our study, and a greater percentage of study participants self-reported having health issues, as this may lower older individuals’ functional level and raise the study’s prevalence rate.

The plausible difference between our study and the study conducted in Lebanon may also be due to sampling techniques. For example, in our study, a multistage sampling technique with more than 10 strata was used. Whereas, in the study conducted in Lebanon, the sample was drawn from only two strata which may result in selection bias. Additionally, the Lebanese study was conducted among rural residents among older people members of the community who suffered from poor financial circumstances and those participants were unable to work as a result of aging will move into urban areas, which may lowered the overall prevalence of that study. On the other hand, the possible explanation for this discrepancy between our study and Japan’s study is the difference in study design and participants’ characteristics. While our investigation used a cross-sectional study design, a prospective cohort study was used in a study conducted in Japan. Unlike our study, the educational level of the majority of the participants in the Japanese study was college and above. As a result, being well-educated enhances the preservation of functional ability because of the increased understanding of medical diagnosis and health care services (26).

This study reported a lower prevalence of functional disability compared with studies done in Tunisia (57.3%) (27), Northeastern India (43.7%) (11), and Haryana India (37.4%) (28). The discrepancy between our study and the Tunisian study may be due to variations in the study setting, which was an institution-based cross-sectional study that included older adults who voluntarily presented with a variety of psychosocial issues. Since a higher proportion of functional disability is found in older adults living in institutions, the prevalence was higher in a Tunisian study.

Similar to this, a multicenter study conducted by Northeastern Indians included institutionalized older adults, who are particularly susceptible to functional disability. On the other hand, our study did not include older adults who were institutionalized. In addition, compared to our study, there was a greater proportion of older people who were 70 years of age or older. As the proportion of older adults increases in this age range, which has led to an increase in the prevalence of functional disability, this age group is at high risk for functional disability (29). In contrast to our study, Indian studies were conducted in rural areas that may have limited access to health services, which results in poorer management and prevention of various medical conditions that may lower the functional level of older adults.

Moreover, factors such as age ≥ 70 years, having comorbidities, being physically inactive, people who developed cognitive impairment, being one or more drug users, and having symptoms of depression were significantly associated with functional disabilities among older adults living in Bahir Dar city.

The odds of developing functional disability in participants whose ages were 70 years and older were 2.24 times more likely as compared with 60–64 years old. The plausible explanation might be that as age advances, there is a deterioration in the integration of multiple physiological systems in our body. As a result, functional capacity may decline, moving from more complex activities to less troublesome ones, considering that carrying out a task requires the combination of different physiological systems (30). In addition to that, as age increases, the immunity systems in the body decrease, so older adults are easily exposed to different diseases that may decline their functional level, which is supported by studies done in India (16), Brazil (13), and Japan (14).

Compared to older adults who were physically active, those who were physically inactive were 2.42 times more likely to develop a functional disability. The possible explanation might be that practicing regular exercise is an important factor in the prevention of functional disability because its effects enhance muscle function, range of motion, balance, and coordination. A study showed that exercise can prevent or delay the development of chronic conditions, increase bone density, encourage social interaction, and prevent the development of symptoms of depression that reduce the occurrence of functional disability (31). This result is consistent with studies conducted in Ireland (32) and India (33).

Participants who were one or more drug users were 3.61 times more likely to develop functional disability than those who did not use. One explanation for this could be that older adults who took two or more drugs had various chronic illnesses, and the side effects of taking multiple medications could lead to repeated poisoning of the liver and kidneys. Currently, the use of many medications is raised, which increases the risk of adverse health outcomes such as increased healthcare expenses, drug–drug interactions, lowered functional status, and various geriatric syndromes that could worsen functional disability in older adults (34). This finding is agreed with a study studied in Ireland (32).

The odds of developing functional disability in participants having comorbidities were 2.96 times higher than those participants who have no comorbidity. The possible explanation could be that having comorbidities develops a greater impact on functional disability and indicates an additive or synergistic effect of various combinations of comorbidities on functional disability (35). Moreover, the presence of multiple chronic diseases may have various complex interactions, causing greater difficulty for older individuals in performing daily activities and requiring assistance from others. This result is consistent with studies conducted in India (36), Korea (37), and Mexico (38).

Older adults who developed cognitive impairment were 3.66 times more likely to develop functional disability than participants who did not develop symptoms of cognitive impairment. The possible explanation could be that as older adults age enhances their concentration and memory decline, which causes the older people to perform various tasks improperly. In addition, older people who have experienced cognitive decline may have deficits in reasoning, memory, perception, attention, and the capacity to understand and recognize personality. Hence, as the decline progresses, dependence and functional disability increase, making it impossible for older people to perform even the most basic daily activities (39). The findings of this study are supported by studies done in Tunisia (40) and Mexico (38).

Furthermore, the odds of developing functional disability in participants who developed symptoms of depression were 1.7 times higher than those who did not develop symptoms of depression. This might be because depressive symptoms may have a negative impact on the quality of life and social relations of older people individuals. Similarly, individuals who have depression symptoms are inactive and have not engaged or participated in different exercises and activities, which will lead them to lose muscle mass and strength. On the other hand, depressive symptoms enhance the decline of physical functioning over time due to the prolonged presence of certain depressive symptoms, symptoms of burden, and negative health behaviors such as physical inactivity, obesity, and non-compliance with various treatment regimens (41). This study is supported by studies conducted in India (36) and Mexico (38).

### Limitations of the study

Because the study employed a cross-sectional study design, it is not possible to determine the causal relationship between functional disability and the variables that have been identified. On the other hand, our study was conducted only in one district and included only urban older adults; the finding of the study is not generalizable to the entire rural population of the country. Furthermore, in this study, variables such as morbidity, cognitive impairment, and depression were assessed through a self-reported questionnaire, which could result in recall bias. Despite these limitations, this study might provide evidence for scholars, clinicians, and other responsible bodies regarding this emerging public health concern in Ethiopia.

## Conclusion

This study found that nearly one-third of the older adults (≥ 60 years) had functional disabilities. Functional disability was significantly associated with factors such as increasing age, presence of comorbidities, being physically inactive, one or more drug users, being cognitively impaired, and being depressed. Proactive measures need to be initiated to ensure proper care and support of functionally disabled individuals, especially targeting vulnerable groups identified in this study.

## Data Availability

The original contributions presented in the study are included in the article/supplementary materials, further inquiries can be directed to the corresponding author.
